# Evaluating Very Deep Convolutional Neural Networks for Nucleus Segmentation from Brightfield Cell Microscopy Images

**DOI:** 10.1177/24725552211023214

**Published:** 2021-06-24

**Authors:** Mohammed A. S. Ali, Oleg Misko, Sten-Oliver Salumaa, Mikhail Papkov, Kaupo Palo, Dmytro Fishman, Leopold Parts

**Affiliations:** 1 Department of Computer Science, University of Tartu, Tartu, Estonia; 2 Ukrainian Catholic University, Lviv, L’vìvs’ka, Ukraine; 3 PerkinElmer Cellular Technologies Germany GmbH, Hamburg, Germany; 4 Wellcome Sanger Institute, Hinxton, Cambridgeshire, UK

**Keywords:** cytometry, image analysis, deep learning, brightfield microscopy

## Abstract

Advances in microscopy have increased output data volumes, and powerful image analysis methods are required to match. In particular, finding and characterizing nuclei from microscopy images, a core cytometry task, remains difficult to automate. While deep learning models have given encouraging results on this problem, the most powerful approaches have not yet been tested for attacking it. Here, we review and evaluate state-of-the-art very deep convolutional neural network architectures and training strategies for segmenting nuclei from brightfield cell images. We tested U-Net as a baseline model; considered U-Net++, Tiramisu, and DeepLabv3+ as latest instances of advanced families of segmentation models; and propose PPU-Net, a novel light-weight alternative. The deeper architectures outperformed standard U-Net and results from previous studies on the challenging brightfield images, with balanced pixel-wise accuracies of up to 86%. PPU-Net achieved this performance with 20-fold fewer parameters than the comparably accurate methods. All models perform better on larger nuclei and in sparser images. We further confirmed that in the absence of plentiful training data, augmentation and pretraining on other data improve performance. In particular, using only 16 images with data augmentation is enough to achieve a pixel-wise F1 score that is within 5% of the one achieved with a full data set for all models. The remaining segmentation errors are mainly due to missed nuclei in dense regions, overlapping cells, and imaging artifacts, indicating the major outstanding challenges.

## Introduction

Microscopes collect images from scales of atoms and molecules to cells and tissues. While visual inspection can guide intuition, automated image processing is central for distilling understanding from the gathered data. Computational analysis approaches have evolved together with the instrumentation, with a plethora of methods developed to date.^1,2^ However, due to the high dimensionality, large data volumes, and complex signals in high-content microscopy, image analysis remains challenging to automate in general.^3^ Perhaps the most important and most thoroughly studied task is identifying nuclei in cell microscopy images,^4^ a common foundational step in many analysis workflows. As generating brightfield images is relatively quick and noninvasive, it would benefit many protocols to be able to segment nuclei directly from them. However, this rewarding task remains challenging^5^ and is therefore the main focus of this work.

Over the last decade, deep learning-based approaches have advanced image classification,^6^ object detection,^7^ and segmentation.^8^ The cell microscopy analysis community and the larger cytometry field in general have taken note and exapted the useful ideas.^9–11^ One of the earliest popular approaches that utilized convolutional neural networks for nuclear segmentation from fluorescence images was DeepCell.^12^ Many more have been proposed since.^13–20^ U-Net,^21^ later superseded by U-Net++,^22^ has also been introduced as a plugin for ImageJ^23^ to make the models accessible for biological image analysis. Overall, classical methods have been outperformed by deep learning techniques for nuclear segmentation, justifying the substantial interest in them.^4^ However, while segmenting the more challenging brightfield cell images has also been attempted,^5,24^ there remains a performance gap compared with fluorescence segmentation.

The rapid development of deep learning has continuously provided new insights that could also impact practical solutions.^8,22,25–27^ The key advances in image segmentation have come from properly accounting for context.^28^ The early approach was to use the features extracted in earlier layers as inputs into deeper ones,^21,29^ followed by considering a broader context for the segmented object.^22,25,30,31^ Training was further improved by data augmentation,^32,33^ and objects of different scales better handled with scale-robust architectures.^30,31^ These recent ideas and advanced networks could improve nuclear segmentation performance as well, but have not yet been utilized for this purpose.

Here, we tackle the nucleus segmentation problem in brightfield and fluorescence images with current state-of-the-art deep learning approaches. We evaluate the architectures of U-Net++,^22^ Deeplabv3+,^30^ Tiramisu,^25^ and a modified version of U-Net,^5^ as well as a novel streamlined PPU-Net architecture, for identifying nuclei. To gain a deeper understanding, we investigate the causes of variable performance across cell lines and images, the sources of error, and the data requirements for training successful segmentation models.

## Materials and Methods

To evaluate modern deep learning methods for the nuclear segmentation task, we use two data sets containing a total of eight different cell lines, five alternative neural network architectures, and a unified training process that includes models with different amounts of training data, transfer learning, label smoothing, data augmentation, and multiple evaluation metrics (Box 1).

**Box 1.** Common definitions.

**Transfer learning:** Applying the knowledge gained from one domain to another one, for example, using a model trained using images from one cell line (source domain) to predict images from another cell line (target domain) **Label smoothing:** Acknowledging possible errors in annotations and setting the prediction target to values away from traditional 0/1 (e.g., 0.1 and 0.9) to reflect this.^42^ **Evaluation metrics:** 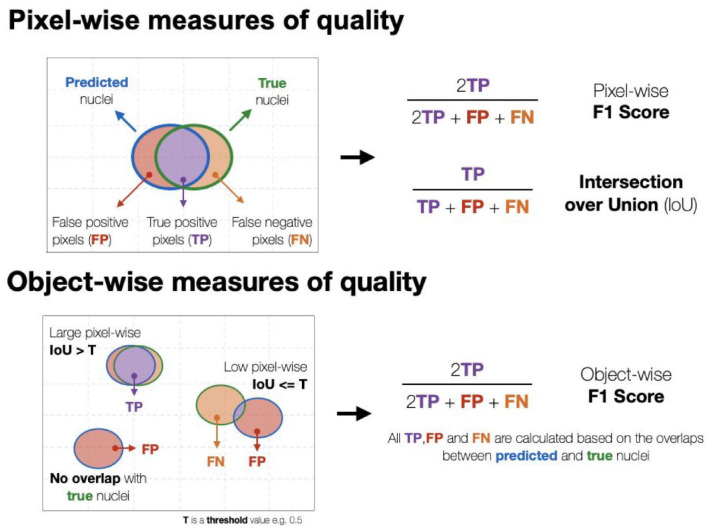

### Network Architectures

We evaluated four convolutional neural networks that cover the successful architectural features for image segmentation: skip connections, atrous convolution, pyramid pooling, and dense blocks. All models are end-to-end trained encoder–decoder networks with a downsampling contraction path, an upsampling expansion path, and a bottleneck to connect them. Inspired by the surveyed literature, we also propose a new architecture (PPU-Net) to strike a balance between model size and performance. We describe each of the models in detail below.

**Figure 1.**
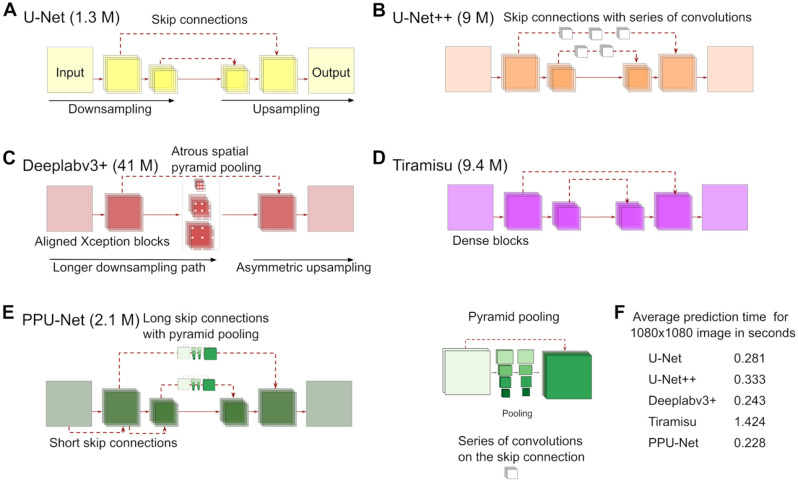
Convolutional neural network architectures. (**A**) U-Net is the baseline model with down- and upsampling paths, as well as standard skip connections. (**B**) U-Net++ introduces a series of convolutions in the skip connection layers. (**C**) Deeplabv3+ uses atrous convolution and spatial pyramid pooling with a simple upsampling path. It uses a modified version of the Xception model35 as its backbone. (**D**) Tiramisu uses a dense block as a core building block. (**E**) The proposed architecture, PPU-Net, applies skip connections between the downsampling blocks and deploys the pyramid pooling module between downsampling and upsampling paths. Brackets next to the model names indicate the number of trainable parameters. (**F**) Average prediction time of a single brightfield image for all models. The experiments were conducted using a Tesla V100-PCIE-16GB Graphics Processing Unit.

As a baseline, the successful U-Net^21^ has already been adapted for brightfield nuclei segmentation.^5^ The architecture has four main components: a contraction path, an expansion path, a bottleneck to connect them, and skip connections to enhance localization by transferring high-resolution features from the contraction to the expansion path (**Fig. 1A**). The contraction path in our implementation contains 15 convolution layers that use 3 × 3 convolution filters followed by rectified linear unit (ReLU)^34^ activation layers, with a 2 × 2 max pooling layer and a skip connection to the upsampling path every third convolution. The expansion path consists of 15 corresponding convolution layers, followed by ReLU activation layers, with an upsampling layer every third convolution. There is a bottleneck block between the encoder and the decoder with three convolution layers. Each convolution layer in the contraction path, expansion path, and bottleneck has 64 filters. In total, the architecture has 1.3 million trainable parameters (**Fig. 1**).

U-Net++^22^ enriches the skip links between the contraction and expansion paths. To achieve this, a series of dense convolutions are added to the encoder feature maps, and their output concatenated to the decoder counterparts (**Fig. 1B**). Both contraction and expansion paths consist of four blocks. Each block consists of two convolution layers, followed by batch normalization, followed by 2 × 2 max pooling in the downsampling path, and transposed convolution for the upsampling path. The bottleneck consists of a similar block, but without max pooling. The skip connection pathways from the first, second, and third contraction path blocks to the corresponding expansion path blocks consist of three, two, and one block, respectively. Each convolution layer in the first block of the contraction path, the corresponding block in the expansion path, and the skip connection pathway between them has 32 filters of size 3 × 3. The second, third, and fourth blocks have 64, 128, and 256 filters, respectively, and the convolution layers in the bottleneck have 512 filters each. In total, U-Net++ has ~9 million trainable parameters (**Fig. 1**).

The Deeplab family of models has evolved to be some of the most sophisticated in the field^8,26,27,30^). Like the other considered models, its most recent member, Deeplabv3+, has the form of an encoder–decoder network, with the Xception backbone^35^ for sharper boundaries of segmented objects. The published encoder module consists of four parts: entry flow, middle flow, exit flow, and atrous spatial pyramid pooling (**Fig. 1C**). The entry, middle, and exit flows have 3, 16, and 2 blocks, respectively, each consisting of two 3 × 3 separable convolutions,^36^ and another one with a stride of 2 for downsampling. The input to each block is concatenated to its output. The decoder branch combines the output of the encoder with feature maps of low-level features. Atrous spatial pyramid pooling uses filters of various sizes for the skip connections. Here, we use the published Deeplabv3+ with the Xception backbone, and the output stride parameter set to 16 to balance performance, accuracy, and speed.^30^ In total, Deeplabv3+ has ~41 million trainable parameters (**Fig. 1**).

The Tiramisu architecture^25^ uses dense blocks^37^ for segmentation (**Fig. 1D**). In a dense block, feature map inputs to each convolutional layer are concatenated to the outputs of all further convolutional layers in that block, introducing deep supervision and feature reuse. The Tiramisu has a contraction path of five dense blocks, each followed by a transition-down block of batch normalization, ReLu, and 2 × 2 max pooling, and an expansion path of five dense blocks, each followed by a transition-up block (3 × 3 transposed convolution with a stride of 2). The dense blocks in the contraction and expansion paths use (4, 5, 7, 10, 12) and (12, 10, 7, 5, 4) layers, respectively. The bottleneck dense block between the two paths contains 15 layers. Finally, a single convolution layer is added to the beginning of the contraction path. Each convolution layer in the dense block has sixteen 3 × 3 filters, while the first convolution layer has forty-eight 3 × 3 filters. In total, this network has ~9.4 million parameters (**Fig. 1**).

Inspired by the diversity of successful ideas in the field, we designed our own pyramid pooling U-Net architecture (PPU-Net) (**Fig. 1E**). Its relevant features attempt to crystalize different aspects of progress in the segmentation models above. First, PPU-Net exploits both short and long skip connections. This was motivated by a demonstration that both types help to achieve better performance and faster convergence; the short skip connections also stabilize parameter updates and help in solving a vanishing gradient problem that prevents parameters from being effectively updated.^32^ Second, to cover the necessary global and subregional context without losing spatial relations, PPU-Net employs the hierarchical pooling module (pyramid pooling^31^) in long skip connections.

The PPU-Net consists of a contraction path, an expansion path, a bottleneck between them, and a skip pathway connection that link the features in the contraction path to the ones in the expansion path. There are 10 blocks in the contraction path, 10 blocks in the expansion path, and 2 blocks in the bottleneck between the paths. A block comprises two 3 × 3 convolutions, each of which is followed by batch normalization and ReLU activation layers. Each convolution layer in such a block has 64 filters. The contraction path block output is processed by the pyramid pooling module in the skip pathway, and concatenated to the input of the corresponding block in the expansion path. This module integrates information from five different scale levels by average pooling the feature maps with pool sizes of 16 × 16, 8 × 8, 4 × 4, 2 × 2, and 1 × 1 and strides that equal the pool size. The output of each pooling level is processed by sixteen 1 × 1 convolutions, followed by batch normalization and ReLU activation layers. The output is rescaled bilinearly to match the input dimensions, and concatenated with the input again (**Fig. 1E**). This architecture is the second smallest after standard U-Net with ~2.1 million trainable parameters, which is 5% of Deeplabv3+, the largest tested architecture. It is also the fastest at segmenting an image, taking 0.23 s, on average (**Fig. 1F**).

### Data

Two data sets were used in this study with a total of eight different cell lines. Their provenance has been described previously,^5^ and we briefly repeat this here. First, human cervical cancer cells (HeLa), epithelial cells from kidney tissue of adult dogs (MDCK), human hepatocellular carcinoma cells (HepG2), human breast cancer cells (MCF7), mouse embryonic fibroblast cells (NIH3T3), human alveolar basal epithelial cells (A549), and human fibrosarcoma (HT1080) were seeded into collagen type 1-coated CellCarrier-384 Ultra Microplates (PerkinElmer, Waltham, MA; cat. 6057700) using 48 wells for each. The cells were fixed in formaldehyde (Sigma, St. Louis, MO; cat. 252549) and stained with 10 µg/mL Hoechst 33342 (Thermo Fisher, Waltham, MA; cat. H3570). Images were acquired using a 20× water immersion objective on an Opera Phenix high-content screening system (PerkinElmer) in confocal mode for both brightfield and fluorescence modalities. A total of 3024 images of size 1080 × 1080 pixels (1 pixel = 0.59 µm) were acquired for each modality, with nine fields of view from each well (432 combined) for each of the seven cell lines, and 353 cells in each field of view, on average (**Suppl. Fig. S1, Suppl. Table S1**). This is referred to as “seven cell line” data.

Second, human prostate adenocarcinoma (LNCaP, sourced from ATCC) cells were seeded into the 384 wells of a CellCarrier Ultra (PerkinElmer) microplate, fixed in formaldehyde, and stained using DRAQ5 fluor (Abcam, Cambridge, United Kingdom) to label nuclear DNA. A total of 784 images of 2556 × 2156 pixels (1 pixel = 0.325 µm) were acquired using a 20× objective in both confocal mode to capture fluorescence images and brightfield mode on a CellVoyager 7000 (Yokogawa, Tokyo, Japan) instrument, giving an average of 681 cells per image (**Suppl. Table S1**). This is referred to as the “LNCaP” data. For both seven cell line and LNCaP data, one modality was acquired first on all wells and fields of view, and the second one in a subsequent round.

Harmony image analysis software (PerkinElmer) with expert quality control to optimize parameters was used to generate ground truth masks from fluorescence images of nuclear stains for the seven cell lines, as described in Fishman et al.^5^ To establish ground truth fluorescence nuclear boundaries for LNCaP, we applied the U-Net++ model previously trained to segment fluorescence micrographs from the seven cell line images ^5^ on these data.

### Training

All the experiments were conducted using a Tesla V100-PCIE-16GB Graphics Processing Unit, and the architectures were built using the Keras framework with TensorFlow backend v1.14.0.^38^

#### Model Comparison

The five models (U-Net, U-Net++, Deeplabv3+, Tiramisu, and PPU-Net) were trained on the seven cell line data set (separately on fluorescence and brightfield images), using 2016 images for training, 504 images for validation, and 504 for testing; and on LNCaP, using 628 images for training, 78 for validation, and 504 for testing. The Adam optimizer^39^ was used to optimize binary cross-entropy loss. Each architecture was trained for up to 500 epochs with (10,000/batch size) iterations. The learning rate was selected as described below, and reduced by a factor of 10 once the validation loss was not improving for 10 consecutive epochs. Training was terminated completely if validation loss was not improving for 20 consecutive epochs. Batches of size 16, 8, 8, 4, and 8 images were used for U-Net, U-Net++, Deeplabv3+, Tiramisu, and PPU-Net networks, respectively, chosen based on the available processing budget.^27,40^ All networks have an input size of 288 × 288 pixels.

#### Learning Rate Selection

Learning rate is among the most critical hyperparameters for training neural networks.^41^ We used the strategy introduced in Smith^40^ to select it separately for each model. We monitored loss during training each model for a few epochs, while gradually increasing the learning rate from a very small value (1e-10) to a very large one (10). The candidate optimal learning rate was manually identified as the value that gives the largest change in loss (**Suppl. Fig. S2**). We then performed full training for the models using different learning rates around the candidate and selected the best. Selected rates for the brightfield and fluorescence data sets are 1e-5, 1e-3, 5e-4, 5e-4, and 5e-4; and 3e-5, 3e-4, 5e-4, 5e-5, and 3e-4 for U-Net, U-Net++, Deeplabv3+, Tiramisu, and PPU-Net, respectively.

#### Effect of Training Data Set Size

To simulate situations in which a data set of a few images is available, different models using an increasingly different number of images were trained. We randomly extracted 1, 2, 4, 8, 16, 32, 64, and 286 images of the A549 cells from the seven cell line data. Those data sets are used to train seven models from each network architecture. The same models are also trained two more times, once with label smoothing and another time with data augmented using five basic data augmentation techniques (horizontal flip, vertical flip, and rotations of 90°, 180°, and 270°). The models were evaluated using the A549 cell line held-out data.

#### Smoothing Factor Selection

In label smoothing, the cross-entropy loss is optimized against soft targets.^42^ The targets were softened such that the positive label 1 is reduced by a smoothing factor and the negative label 0 is increased by the same factor. We selected the best smoothing factor by conducting a grid search on models trained using factors of 0.05, 0.10, 0.15, and 0.20; selected the best performing of each architecture; and evaluated them on held-out data (**Suppl. Table S2**). The selected factors are 0.05, 0.15, 0.20, 0.20, and 0.15 for U-Net, U-Net++, Deeplabv3+, Tiramisu, and PPU-Net, respectively.

#### Effect of Architecture Selection

To test the effect of making different selections for network architecture, we evaluated three aspects that distinguish U-Net from U-Net++. First, we doubled the number of U-Net pathway connections. Second, we added batch normalization^33^ to the vanilla U-Net. Third, we increased the number of connections as well as introduced batch normalization, and further modified the number of filters in the convolution layers. We gradually increased the number of filters in the contraction path to be 64, 128, 256, and 512 filters while decreasing them symmetrically in the expansion path. Finally, we excluded half of the U-Net++ convolutions in the skip connection layers.

### Transfer Learning

To evaluate the models’ performance across domains, we fine-tuned a model trained on a source domain (one data set) using images from the target domain (another data set), as well as using images from both source and target domains. In both cases, we used an increasing number of images (1, 2, 4, 8, 16, 32, 64, and 128) to fine-tune the model. When fine-tuning on images from multiple domains, the same number was picked from both domains. We examined two sets of source and target domains. First, we used six cell lines from the seven cell line data set as the source domain and the remaining cell line as the target domain, and repeated for each cell line, introducing a small domain shift of the different line, but still considering images from the same acquisition experiment. Second, we used the LNCaP data set as a source domain and the seven cell line data collected on another instrument as a target domain, introducing a large domain shift of the imaging instrumentation and laboratory undertaking the work. We conducted the experiments of this section on the lightest model (U-Net), as we expect domain shift, rather than model differences, to dominate quality.

### Effect of Number of Training Focal Planes

The LNCaP data set was used to learn about the impact on segmentation performance with different numbers of unique focal planes as network input. To keep the model’s number of parameters constant, the number of input channels was fixed to nine, and the number of input focal planes was varied from nine copies of a single plane to nine different planes. The order in which to add planes was experimentally determined. First, we trained a different model for each plane and picked the best input plane based on evaluation. Then, we repeated the experiment to pick the next best plane out of eight possible variants in addition to the previously picked one.

### Postprocessing and Evaluation

To evaluate models, we first postprocessed the image probability maps they produce. All results are based on pixel-level outputs binarized at a 0.5 cutoff unless detailed otherwise. Objects are detected by clustering the interconnected positive pixels into objects using measure.label from the skimage package.^43^ We filtered out objects smaller than 25 pixels and filled out holes smaller than 25 pixels using remove_small_objects and remove_small_holes, respectively, from the same package.

We used both pixel-wise and object-wise metrics (Box 1) to quantify model performance. The accuracy and F1 score used for pixels are standard in machine learning.^5^ To quantify object-level accuracy, we measure the intersection over union (number of pixels in intersection of two objects divided by number of pixels in their union [IoU]) for pairs of segmented and ground truth objects, and consider a ground truth object detected at an IoU threshold if there is a segmented object with an IoU value to it above the threshold. We report the F1 score for object detection across IoU thresholds ranging from 0.5 to 0.95 with a step of 0.05, as well as averaged over these thresholds (object-wise F1 score), as established earlier.^4^ We also record the number of merges (more than one ground truth object overlaps a predicted one), splits (a single object in the ground truth overlaps multiple predicted objects), and missed objects (ground truth objects that are not detected). To detect splits and merges, which lead to a small object overlap by definition, we used an IoU threshold of 0.1. To detect missed objects, we used an IoU threshold of 0.6, which gives a good balance between strict overlap and not missing objects entirely.

Finally, we assigned a likely cause of error to mislabeled pixels. First, we assigned errors to noisy input data if at least four out of five models agree on the same prediction in a fluorescence image that is opposite to the ground truth label, and calculate the fraction of errors that these pixels account for. Second, to quantify the error contributed by imaging artifacts, we manually annotated artifacts in 20 images and recalculated performance outside those anomalous regions to derive a difference in error that can then be ascribed to the artifacts.

## Results

**Figure 2.**
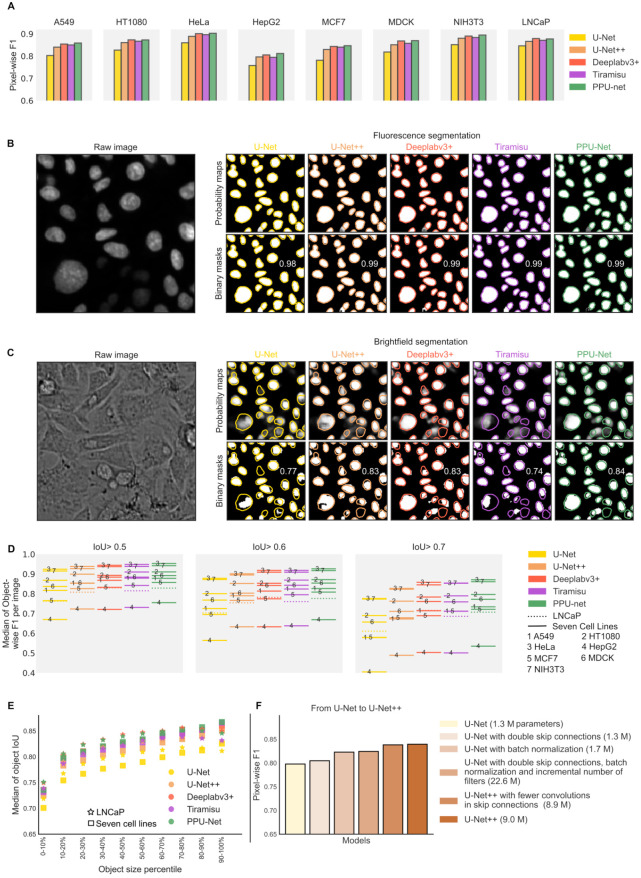
Comparison of models’ performance. (**A**) Pixel-wise F1 scores (*y* axis) for models (colors) trained on brightfield images of all seven cell lines (left seven subplots) and the LNCaP data set (rightmost subplot). (**B,C**) Example of matched fluorescence (**B**) and brightfield (**C**) images and the corresponding segmentation from all models (right; *x* axis of panels). Top panels: Probability maps. Bottom panels: Maps binarized at the 0.5 threshold. Inscribed number, pixel-wise F1 score for the corresponding model; colored contours, ground truth nucleus boundaries. (**D**) Per-image median object-wise F1 scores (*y* axis) across seven cell lines (solid lines) and LNCaP (dotted lines) for all models (colors) and a range of IoU thresholds (panels; Material and Methods). (**E**) Median of object IoU scores in all seven cell line and LNCaP data sets (*y* axis, markers) for each decile of object sizes (*x* axis) for each model (colors). (**F**) Pixel-wise F1 score (*y* axis) of the baseline model (U-Net, yellow), U-Net++ (brown), and their architecture modifications (*x* axis, colors; Materials and Methods) on the A549 cell line. Brackets in the legend indicate the number of trainable parameters in millions.

We assessed the performance of four state-of-the-art and one novel segmentation model on the seven cell line and LNCaP data sets (**Fig. 2, Suppl. Tables S3 and S4**). All models detected nuclei in the fluorescence images with high pixel-wise F1 scores (average over all seven cell lines, 99% for U-Net, 99% for U-Net++, 98% for Deeplabv3+, 99% for Tiramisu, and 99% for PPU-Net) (**Suppl. Table S3; Box 1**), as well as object-wise scores (96%, 97%, 95%, 97%, and 97% respectively) (**Suppl. Table S3**). This confirms that the signal in the fluorescence channel is clear enough to be detected regardless of the model used (**Fig. 2B**), as has been observed before.^4,5^ We therefore consider fluorescence segmentation a solved problem to a practical limit, and the rest of the results focus on the more challenging brightfield modality.

## Model Performance on Brightfield Images

Unlike for fluorescence images, models’ performances on brightfield images have a wider range. The methods achieved pixel-wise F1 scores of 81.3%, 85.0%, 86.2%, 85.6%, and 86.5% for U-Net, U-Net++, Deeplabv3+, Tiramisu, and PPU-Net, respectively (**Fig. 2A, Suppl. Table S3**) in the seven cell line data set; and 84.0%, 86.2%, 87.7%, 85.8%, and 87.4% in the LNCaP data set, demonstrating that the classical U-Net model is outperformed by newer architectures (**Fig. 2A–C**). This trend is consistent across individual images (**Suppl. Fig. S3**) as well as within specific cell lines, and therefore is likely due to real performance differences, rather than biases from individual images or cell lines.

Next, we assessed object segmentation performance, using the area intersection over union (“IoU,” Materials and Methods, Box 1^4^) to identify correctly segmented nuclei. In line with pixel-wise results, PPU-Net slightly outperformed other models in the seven cell lines and was on a par with Deeplabv3+ in the LNCaP data set. Classical U-Net was inferior (object-wise F1 scores of 48.8%, 55.4%, 58.1%, 58.2%, and 59.8% for U-Net, U-Net++, Deeplabv3+, Tiramisu, and PPU-Net, respectively, for seven cell lines; and 45.2%, 51.0%, 54.9%, 51.4%, and 54.6% for the LNCaP data set) (**Suppl. Fig. S4**). Importantly for cytometry applications, we confirmed that the object detection quality is echoed in the ability to recapitulate object properties. Indeed, a higher object-wise F1 score also gives a better match to ground truth object solidity and size (**Suppl. Fig. S6**).

## Data Set and Architecture Properties Influencing Performance

It is important to understand when brightfield segmentation can be expected to be successful and what the quality determining factors are. We first tested whether cell density influences segmentation performance and found a negative correlation between the number of cells per image and the pixel-wise F1 score (Pearson’s *R* = −0.65, −0.66, −0.69, −0.67, and −0.70 for U-Net, U-Net++, Deeplabv3+, Tiramisu, and PPU-Net in seven cell line data, respectively; and Pearson’s *R* = −0.39, −0.39, −0.46, −16, and −0.40 for the same respective models in the LNCap data). This trend held across all data, as well as within images from individual cell lines (**Suppl. Fig. S7**), with the exception of the MDCK line, which has a narrow range of low cell densities. Regression line slopes per model ranged between −1.2 × 10^–4^ and −1.4 × 10^–4^, suggesting that the models did not differ substantially in the degradation of their performance as cell densities decreased. Overall, dense images are more challenging to segment into individual objects, as expected.

Next, we considered whether the size of nuclei impacts the accuracy of prediction. We observed that larger objects are segmented more accurately by all models in the complete data set (**Fig. 2E, Suppl. Fig. S8**). The objects in the largest decile have at least a 9% greater median pixel-wise F1 score than the objects in the smallest decile for all models in both the seven cell line and LNCaP data sets (**Fig. 2E**). We also wondered whether models cope differently with images that have variability in object size, but saw no effect (Pearson’s *R* values range from 0.03 to 0.08) (**Suppl. Fig. S9**).

As we found models to have a range of performances, we attempted to understand which model features are responsible for the differences. We suspected that the lower performance of U-Net could be ascribed to the model representation capacity (e.g., number of trainable parameters) and training approach. To test this, we evaluated three aspects that distinguish it from the otherwise similar U-Net++ architecture. First, we doubled the U-Net pathway connections, and this improved the pixel-wise F1 score by 0.006 (**Fig. 2F**). Second, we added batch normalization,^33^ which also improved the pixel-wise F1 score by 0.02. We then increased the number of connections as well as introduced batch normalization, and further modified the number of filters in the convolution layers, improving the score by 0.026 compared with baseline. Finally, we excluded half of the U-Net++ convolutions in the skip connection layers but did not observe substantial deterioration in performance (0.001 in pixel-wise F1 score) (**Fig. 2F**). These results suggest that multiple architecture choices are responsible for the largest observed model performance differences, and that changing the number of parameters alone is not sufficient to achieve large changes to performance.

### Common Errors in Segmentation

**Figure 3.**
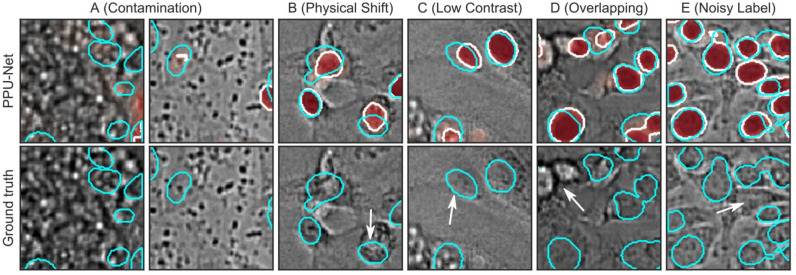
Examples of errors from visual inspection. Cyan, ground truth contour; white, prediction contour; red, prediction probability maps; white arrows, error. Top images: Ground truth and PPU-net model predictions. Bottom images: Ground truth only. (**A**) Severe contamination (left) and mild contamination (right). (**B**) Physical nucleus shift between fluorescence and brightfield modalities. (**C**) Low contrast. (**D**) Overlapping between out-of-focus and in-focus nuclei. (**E**) Noisy ground truth.

The results of the second-generation deep learning models for brightfield nuclei detection were better than earlier reports,^4,5^ but errors still occurred. We next visually inspected output segmentations and found that the errors are mainly due to four effects. First, some samples were contaminated (**Fig. 3A**). Based on the severity of the contamination, all models struggle to delineate a nucleus and can miss it entirely. We observed likely cases of biological contamination during cell culture (e.g., bacteria) (**Fig. 3A**, right panel), as well as particle contamination during acquisition (e.g., dandruff or skin) (**Fig. 3A**, left panel). Second, predicted nucleus boundaries are shifted between brightfield and fluorescence modalities (**Fig. 3B**). We confirmed visually that the image registration was correct overall, and other cells in this field of view are concordant, and therefore hypothesize that the mismatch is due to a true physical shift between brightfield and fluorescence modalities, for example, if cells are moving when not properly adhered to the plate. Third, there can be low contrast between the cell nuclei and the background (**Fig. 3C**). This lack of signal can lead models to miss nuclei or to merge objects. Finally, out-of-focus cells can still be visible in fluorescence images, but are difficult to detect in brightfield, as their signal is distorted (**Fig. 3D**). These findings are consistent with error modes previously characterized in these data.^5^

We next attempted to quantify the relative contribution of the various errors. To do so, we first picked 20 images with evidence of sample contamination and recalculated performance outside of manually annotated anomalous regions only. An average of 10.7% (range, 9.3%–11.8%) of misclassified pixels were caused by those anomalies, and filtering out the anomalous regions improved the pixel-wise F1 score by 1.6%–1.9% per image. This suggests that artifacts are a substantial but not the major source of remaining pixel errors in segmentation. Second, we found that 53%–61% of errors are due to false-negative pixels, consistent with either underprediction at object boundaries or missing entire objects, contributing more errors compared with false-positive pixels. Finally, we estimate 3%–5% of misclassified pixels to be due to noise in the ground truth labels (**Fig. 3E**). Together, these results suggest that anomalies and noisy labels contribute about 15% of the errors.

Next, we considered object-level errors of splits, merges, and missing nuclei at a range of pixel classification thresholds (Materials and Methods). Compared with other advanced models, Tiramisu had the smallest number of total merges (5376 in seven cell lines, 4182 in LNCaP) and splits (4271 in seven cell lines, 3088 in LNCaP), on average, considering 176,946 and 53,828 objects in the seven cell line and LNCaP data sets, respectively (**Suppl. Fig. S10**). However, it also had a large proportion of undetected nuclei, on average, across all the thresholds (35% in both data sets) (example in **Fig. 2C**). Taking into account all types of errors and requiring a stricter object overlap, PPU-Net had the best object-wise performance by a narrow margin over Tiramisu and Deeplabv3+ in the seven cell line data set, with F1 scores of 59.8%, 58.2%, and 58.1%, respectively, and U-Net++ and U-Net having lower scores of 55.4% and 48.8% (**Suppl. Fig. S4**). In the LNCaP data set, PPU-Net and Deeplabv3+ had on-par performance data with object-wise F1 scores of 54.6% and 54.9%, respectively.

### Training Choices Influencing Performance

**Figure 4.**
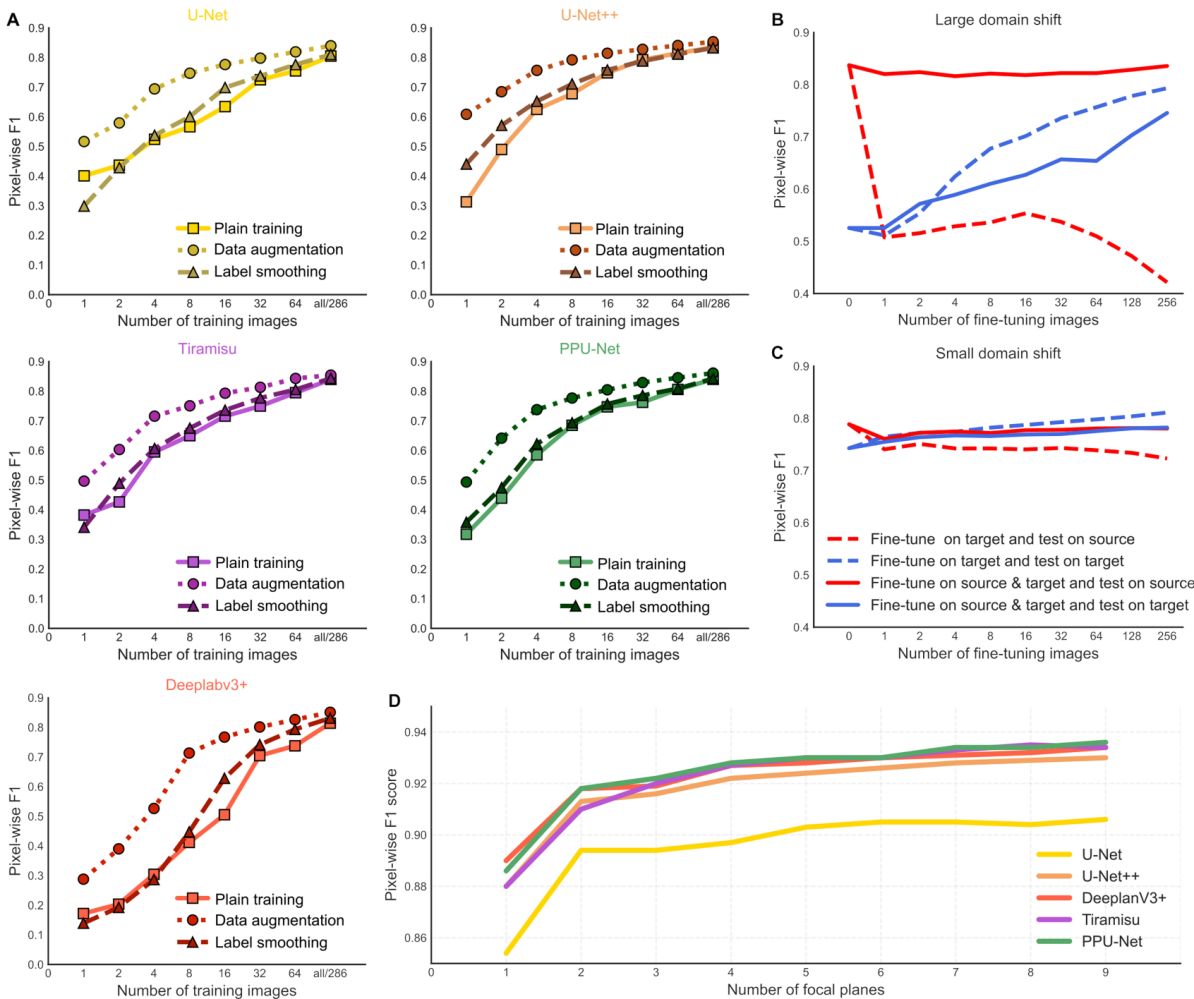
More training data from a relevant domain improves accuracy. (**A**) Pixel-wise F1 score on the A549 cell line (*y* axis) for models trained plainly (solid line), with label smoothing (dashed line) or with data augmentation (dotted line) for U-Net, U-Net++, Deeplabv3+, Tiramisu, and PPU-Net (panels, colors) for an increasing number of training images (*x* axis). (**B**) Pixel-wise F1 score for the U-Net model on the A549 cell line (*y* axis) for an increasing number of training images (*x* axis), fine-tuning on the target domain (dashed line) or source and target domains (solid line) and testing on the source domain (red line) or target domain (blue line). Source domain of six of seven cell lines in the seven cell line data set; target domain the seventh cell line. (**C**) As in **B**, but using the source domain of the LNCaP data set and target domain of the seven cell line data set. (**D**) Pixel-wise F1 scores (*y* axis) for all models (colors) for an increasing number of focal planes (*x* axis) used as input during training.

The results so far were obtained on a large training data set that has thousands of annotated images. In practice, annotation is expensive, and a limited number of annotated images are available. Hence, an important practical question, especially for advanced architectures with millions of parameters, is how many training images are sufficient for optimal performance. Predictably, the model performance improves with more annotated images (**Fig. 4A**).

We tested whether label smoothing (using soft targets in ground truth masks, for example, 0.1 and 0.9 instead of 0 and 1^42^) and data augmentation improve performance under limited training data. We found that nearly all models performed better using each of those strategies compared with training models without any of them (**Fig. 4A**). Deeplabv3+ did not perform well given a small number of training images using standard or label smoothing strategies, which can be due to its large number of parameters (**Fig. 1**), but other models improved performance by 0.5%–2%, on average, when label smoothing was used. Data augmentation improves the performance of all models more, with an average increase in pixel-wise F1 score from 9% to 11%. Moreover, using only 16 images with data augmentation is enough to achieve a pixel-wise F1 score that is within 5% of the one achieved with a full data set for all models. Improvements provided by both strategies diminish when the data set size increases.

Next, we considered transfer learning to deal with limited training data. First, we used a model that was trained on one data set (source domain) and to segment images in another (target domain). Intuitively, the performance then indicates how distant the target and source domains are. None of the transferred models perform near the optimum in another domain, and the domain shift is most marked for cells from another imaging experiment on different instrumentation (31% performance gap when source and target domains are LNCaP and seven cell lines, respectively) (**Fig. 4B**). Conversely, a model trained on a subset of six of the seven cell lines in one imaging experiment, and applied on the seventh, only performed 5% worse than the best one trained on that cell line (**Fig. 4C**).

Next, we used an increasing number of images from the target domain to fine-tune the model. Fine-tuning improves performance on the target domain but degrades performance on the source domain (**Fig. 4B,C**). To avoid the loss of performance on the source domain, we then fine-tuned the model using data from both domains. This retained F1 scores within 2% of the optimum on the source while improving the score on the target domain as the number of fine-tuning images was increased (**Fig. 4B,C**). Therefore, if the goal is to use the model in different domains, training data should be maintained for all of them, and the fine-tuning data set should reflect this.

Finally, we asked whether increasing the number of focal planes that are used in training improves segmentation performance. We found that adding one additional plane increased the pixel-wise F1 score by 3.3% on average, ranging from 4% in U-Net to 2.8% in DeeplabV3+ (**Fig. 4D**). Additional planes gave diminishing returns, with scores within 1.7% of the two-plane performance, on average.

## Discussion

We have surveyed the literature for developments in deep learning for segmentation and evaluated most advanced examples of multiple model families for their ability to identify nuclei in fluorescence and brightfield cell images. We found that models range from moderately performing (U-Net) to well-performing (Tiramisu, U-Net++, and Deeplabv3+) ones, and proposed PPU-Net, a novel architecture for this task. PPU-Net segments nuclei as accurately as the comparable alternative models while featuring smaller size, shorter training time, and quicker prediction. We noticed that the performance of complex models like Deeplabv3+ degrades when the amount of training data is small. We identified the number of focal planes, cell density, and nuclear size (but not its variability across cells), to influence segmentation quality, and established that a small number of ground truth images combined with substantial augmentation is sufficient for training a well-performing model. To our knowledge, these are the first experiments to segment nuclei from brightfield cell microscopy images with very deep neural networks, novel insights into their performance, and the most accurate segmentations presented to date.

The second generation of deep learning models for brightfield nuclei detection were superior to the initial tests on the same data,^5^ but not yet as accurate as fluorescence-based segmentation approaches. Part of this improvement can be ascribed to advances in methodology, where the network size, qualitative features, and training approaches all had an effect on the outcome quality. In concordance with prior work, we observed modality-specific error sources, such as low contrast, likely physical shifts, and noisy ground truth labels. Some of these, such as physical shift, are systematic and unlikely to be improved by more complex models. Others, like out-of-focus cells, could be optimized by dedicated data acquisition and training. Upon inspecting the errors, we believe that there is room for further improvement to the current models, mainly by avoiding anomalous regions, having noisy labels in training data, and better segmentation of smaller objects.

As expected, and observed before (e.g., Caicedo et al.^4^ and Fishman et al.^5^), providing more training data improves the ability to accurately identify nuclei. While data acquisition is not limiting, annotating ground truth in brightfield modality can be a substantial bottleneck, even when fluorescence-guided nuclear segmentation is available. Various data augmentation techniques, such as signal-preserving orthogonal rotations and reflections, as well as lossy general rotations and scalings, can all help bootstrap additional signal for the same data, and thereby improve training for models that do not take these invariants into account. Soft labeling, or intuitively allowing false-positive and false-negative rates in the ground truth, also improves outcomes. Therefore, when compute time and cost are not limiting, but data set sizes are, we recommend augmenting the training data.

Our new segmentation architecture, PPU-Net, is arguably the most practical. While the U-Net model is the smallest, its performance was dominated by the larger models with additional features. For example, it has been demonstrated that the residual connections, as employed in Tiramisu and shown in Drozdzal et al.,^32^ give a substantial performance boost and stable training. Inspired by these networks, PPU-Net similarly employs simple light residual connections and achieves good performance in both brightfield and fluorescence modalities. Its smaller size and faster speed make PPU-Net more suitable to use in large-scale experiments.

Performance was variable across objects of different size and density. The large nuclei were well segmented in general. This could be due to technical reasons (they have more pixels, and therefore a smaller fraction of the area close to the more variable border), additional signal (more photons inform of their location), or a simpler context (larger nuclei also have larger cells, separating them from neighboring nuclei by a bigger distance). Conversely, the most difficult nuclei to segment were small and densely packed, in particular, for the HepG2 cell line. The dense packing problem is a general standing issue in instance segmentation. Dedicated object delineation models and bespoke data sets outside the scope of this work are needed to establish the best way of attacking this in cell microscopy.

The quality of brightfield cell nucleus segmentations is such that they are useful in practice. A major future direction is to expand this approach to segment entire cells, which would aid cytometry applications, especially in cases of relatively dense cultures. Substantial additional training data, as well as innovation in handling dense and overlapping objects, are required to make progress in this direction.

## Supplemental Material

sj-pdf-1-jbx-10.1177_24725552211023214 – Supplemental material for Evaluating Very Deep Convolutional Neural Networks for Nucleus Segmentation from Brightfield Cell Microscopy Images Click here for additional data file. Supplemental material, sj-pdf-1-jbx-10.1177_24725552211023214 for Evaluating Very Deep Convolutional Neural Networks for Nucleus Segmentation from Brightfield Cell Microscopy Images by Mohammed A. S. Ali, Oleg Misko, Sten-Oliver Salumaa, Mikhail Papkov, Kaupo Palo, Dmytro Fishman and Leopold Parts in SLAS Discovery

## References

[1] Angermueller C. Pärnamaa T. Parts L., et al. Deep Learning for Computational Biology. Mol. Syst. Biol. 2016, 12, 878. 27474269 10.15252/msb.20156651 PMC4965871

[2] Bolón-Canedo V. Remeseiro B. Feature Selection in Image Analysis: A Survey. Artif. Intell. Rev. 2020, 53, 2905–2931.

[3] Gómez-de-Mariscal E. Maška M. Kotrbová A., et al. Deep-Learning-Based Segmentation of Small Extracellular Vesicles in Transmission Electron Microscopy Images. Sci. Rep. 2019, 9, 13211. 31519998 10.1038/s41598-019-49431-3 PMC6744556

[4] Caicedo J. C. Roth J. Goodman A., et al. Evaluation of Deep Learning Strategies for Nucleus Segmentation in Fluorescence Images. Cytometry A 2019, 95, 952–965. 31313519 10.1002/cyto.a.23863 PMC6771982

[5] Fishman D. Salumaa S.-O. Majoral D., et al. Segmenting Nuclei in Brightfield Images with Neural Networks. bioRxiv 2019. DOI: 10.1101/764894.

[6] Krizhevsky A. Sutskever I. Hinton G. E. ImageNet Classification with Deep Convolutional Neural Networks. Commun. ACM 2017, 60, 84–90.

[7] Redmon J. Divvala S. Girshick R., et al. You Only Look Once: Unified, Real-Time Object Detection. In 2016 IEEE Conference on Computer Vision and Pattern Recognition, Las Vegas, NV, June 20, 2016; pp 779–788.

[8] Chen L.-C. Papandreou G. Kokkinos I., et al. DeepLab: Semantic Image Segmentation with Deep Convolutional Nets, Atrous Convolution, and Fully Connected CRFs. IEEE Trans. Pattern Anal. Mach. Intell. 2018, 40, 834–848. 28463186 10.1109/TPAMI.2017.2699184

[9] Jones W. Alasoo K. Fishman D., et al. Computational Biology: Deep Learning. Emerg. Top. Life Sci. 2017, 1, 257–274. 33525807 10.1042/ETLS20160025 PMC7289034

[10] Moen E. Bannon D. Kudo T., et al. Deep Learning for Cellular Image Analysis. Nat. Methods 2019, 16, 1233–1246. 31133758 10.1038/s41592-019-0403-1 PMC8759575

[11] Madabhushi A. Lee G. Image Analysis and Machine Learning in Digital Pathology: Challenges and Opportunities. Med. Image Anal. 2016, 33, 170–175. 27423409 10.1016/j.media.2016.06.037 PMC5556681

[12] Van Valen D. A. Kudo T. Lane K. M., et al. Deep Learning Automates the Quantitative Analysis of Individual Cells in Live-Cell Imaging Experiments. PLoS Comput. Biol. 2016, 12, e1005177. 10.1371/journal.pcbi.1005177 PMC5096676 27814364

[13] Xie L. Qi J. Pan L., et al. Integrating Deep Convolutional Neural Networks with Marker-Controlled Watershed for Overlapping Nuclei Segmentation in Histopathology Images. Neurocomputing 2020, 376, 166–179.

[14] Sornapudi S. Stanley R. J. Stoecker W. V., et al. Deep Learning Nuclei Detection in Digitized Histology Images by Superpixels. J. Pathol. Inform. 2018, 9, 5. 29619277 10.4103/jpi.jpi_74_17 PMC5869967

[15] Al-Kofahi Y. Zaltsman A. Graves R., et al. A Deep Learning-Based Algorithm for 2-D Cell Segmentation in Microscopy Images. BMC Bioinformatics 2018, 19, 365. 30285608 10.1186/s12859-018-2375-z PMC6171227

[16] Sirinukunwattana K. Raza S. E. A. Tsang Y.-W., et al. Locality Sensitive Deep Learning for Detection and Classification of Nuclei in Routine Colon Cancer Histology Images. IEEE Trans. Med. Imaging 2016, 35, 1196–1206. 26863654 10.1109/TMI.2016.2525803

[17] Naylor P. Lae M. Reyal F., et al. Nuclei Segmentation in Histopathology Images Using Deep Neural Networks. In 2017 IEEE 14th International Symposium on Biomedical Imaging, Iowa City, IA, April 18–21, 2017; pp 933–936.

[18] Vuola A. O. Akram S. U. Kannala J. Mask-RCNN and U-Net Ensembled for Nuclei Segmentation. In 2019 IEEE 16th International Symposium on Biomedical Imaging, Venice, Italy, April 8–11, 2019; pp 208–212.

[19] Hollandi R. Szkalisity A. Toth T., et al. NucleAIzer: A Parameter-Free Deep Learning Framework for Nucleus Segmentation Using Image Style Transfer. Cell Syst. 2020, 10, 453–458.e6. 10.1016/j.cels.2020.04.003 PMC8247631 34222682

[20] Kromp F. Fischer L. Bozsaky E., et al. Deep Learning Architectures for Generalized Immunofluorescence Based Nuclear Image Segmentation. arXiv 2019. arXiv:1907.12975.

[21] Ronneberger O. Fischer P. Brox T. U-Net: Convolutional Networks for Biomedical Image Segmentation. In 18th International Conference on Medical Image Computing and Computer-Assisted Intervention, Munich, Germany, Oct 2015; pp 234–241.

[22] Zhou Z. Siddiquee M. M. R. Tajbakhsh N., et al. Unet++ : A Nested U-Net Architecture for Medical Image Segmentation. In 4th International Workshop on Deep Learning in Medical Image Analysis and Multimodal Learning for Clinical Decision Support, Granada, Spain, Sept 20, 2018; pp 3–11. 10.1007/978-3-030-00889-5_1 PMC7329239 32613207

[23] Falk T. Mai D. Bensch R., et al. U-Net: Deep Learning for Cell Counting, Detection, and Morphometry. Nat. Methods 2019, 16, 67–70. 30559429 10.1038/s41592-018-0261-2

[24] Sadanandan S. K. Ranefall P. Le Guyader S., et al. Automated Training of Deep Convolutional Neural Networks for Cell Segmentation. Sci. Rep. 2017, 7, 7860. 28798336 10.1038/s41598-017-07599-6 PMC5552800

[25] Jegou S. Drozdzal M. Vazquez D., et al. The One Hundred Layers Tiramisu: Fully Convolutional DenseNets for Semantic Segmentation. In 2017 IEEE Conference on Computer Vision and Pattern Recognition, Honolulu, HI, July 21–26, 2017; pp 11–19.

[26] Chen L.-C. Papandreou G. Kokkinos I., et al. Semantic Image Segmentation with Deep Convolutional Nets and Fully Connected CRFs. IEEE Trans. Pattern Anal. Mach. Intell. 2017, 40, 834–848. 28463186 10.1109/TPAMI.2017.2699184

[27] Chen L.-C. Papandreou G. Schroff F., et al. Rethinking Atrous Convolution for Semantic Image Segmentation. arXiv 2017. https://www.arxiv-vanity.com/papers/1706.05587/ (accessed June 6, 2021).

[28] Lateef F. Ruichek Y. Survey on Semantic Segmentation Using Deep Learning Techniques. Neurocomputing 2019, 338, 321–348.

[29] Noh H. Hong S. Han B. Learning Deconvolution Network for Semantic Segmentation. In 2015 IEEE International Conference on Computer Vision, Santiago, Chile, Dec 7–13, 2015; 1520–1528.

[30] Chen L.-C. Zhu Y. Papandreou G., et al. Encoder-Decoder with Atrous Separable Convolution for Semantic Image Segmentation. In 15th European Conference on Computer Vision, Munich, Germany, Sept 8–14, 2018; pp 833–851.

[31] Zhao H. Shi J. Qi X., et al. Pyramid Scene Parsing Network. In 2017 IEEE Conference on Computer Vision and Pattern Recognition, Honolulu, HI, July 21–26, 2017; pp 2881–2890.

[32] Drozdzal M. Vorontsov E. Chartrand G., et al. The Importance of Skip Connections in Biomedical Image Segmentation. In Deep Learning and Data Labeling for Medical Applications, Athens, Greece, Oct 21, 2016; pp 179–187.

[33] Ioffe S. Szegedy C. Batch Normalization: Accelerating Deep Network Training by Reducing Internal Covariate Shift. In 32nd International Conference on Machine Learning, Lille, France, July 6–11, 2015; pp 448–456.

[34] Nair V. Hinton G. E. Rectified Linear Units Improve Restricted Boltzmann Machines. In 27th International Conference on Machine Learning, Haifa, Israel, June 21–24, 2010; pp 807–814.

[35] Qi H. Zhang Z. Xiao B., et al. Deformable Convolutional Networks—Coco Detection and Segmentation Challenge 2017 Entry. In ICCV COCO Challenge Workshop, Vol. 15, 2017; pp 764–773.

[36] Vanhoucke V. Learning Visual Representations at Scale. ICLR Invited Talk, April 16, 2014. https://www.youtube.com/watch?v=VhLe-u0M1a8 (accessed June 5, 2021).

[37] Huang G. Liu Z. Van Der Maaten L., et al. Densely Connected Convolutional Networks. In 2017 IEEE Conference on Computer Vision and Pattern Recognition, Honolulu, HI, July 21–26, 2017; pp 4700–4708.

[38] Abadi M. Barham P. Chen J., et al. TensorFlow: A System for Large-Scale Machine Learning. In 12th USENIX Symposium on Operating Systems Design and Implementation, Savannah, GA, Aug 22, 2016; pp 265–283.

[39] Kingma D. P. Ba J. Adam: A Method for Stochastic Optimization. arXiv 2014. arXiv:1412.6980.

[40] Smith L. N. A Disciplined Approach to Neural Network Hyper-Parameters: Part 1—Learning Rate, Batch Size, Momentum, and Weight Decay. arXiv 2018. arXiv:1803.09820.

[41] Goodfellow I. Bengio Y.; Courville, A. Deep Learning, Vol. 1, No. 2. MIT Press: Cambridge, 2016.

[42] Szegedy C. Vanhoucke V. Ioffe S., et al. Rethinking the Inception Architecture for Computer Vision. In 2016 IEEE Conference on Computer Vision and Pattern Recognition, Las Vegas, NV, June 20, 2016; pp 2818–2826.

[43] van der Walt S. Schönberger J. L. Nunez-Iglesias J., et al. scikit-image: Image Processing in Python. PeerJ 2014, 2, e453. 10.7717/peerj.453 PMC4081273 25024921

