# Supplementary material for: Evaluating Very Deep Convolutional Neural Networks for Nucleus Segmentation from Brightfield Cell Microscopy Images
Source: SLAS Discov. 2021 Jun 24;26(9):1125–37. doi: 10.1177/24725552211023214 (PMC8458686; doi:10.1177/24725552211023214)
Supplement: sj-pdf-1-jbx-10.1177_24725552211023214 – Supplemental material for Evaluating Very Deep Convolutional Neural Networks for Nucleus Segmentation from Brightfield Cell Microscopy Images [file sj-pdf-1-jbx-10.1177_24725552211023214.pdf]

I. Supplementary Material

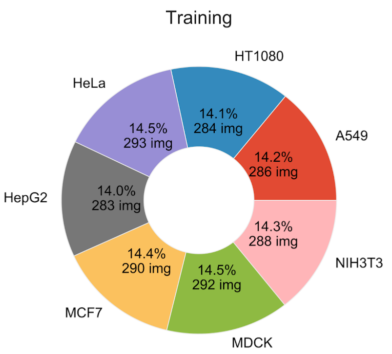

Figure S1: Distribution of seven cell line dataset into training (left), validation (middle), and test (right) splits. In each split, the fraction and number of images from each cell line are shown (pie chart; colors).

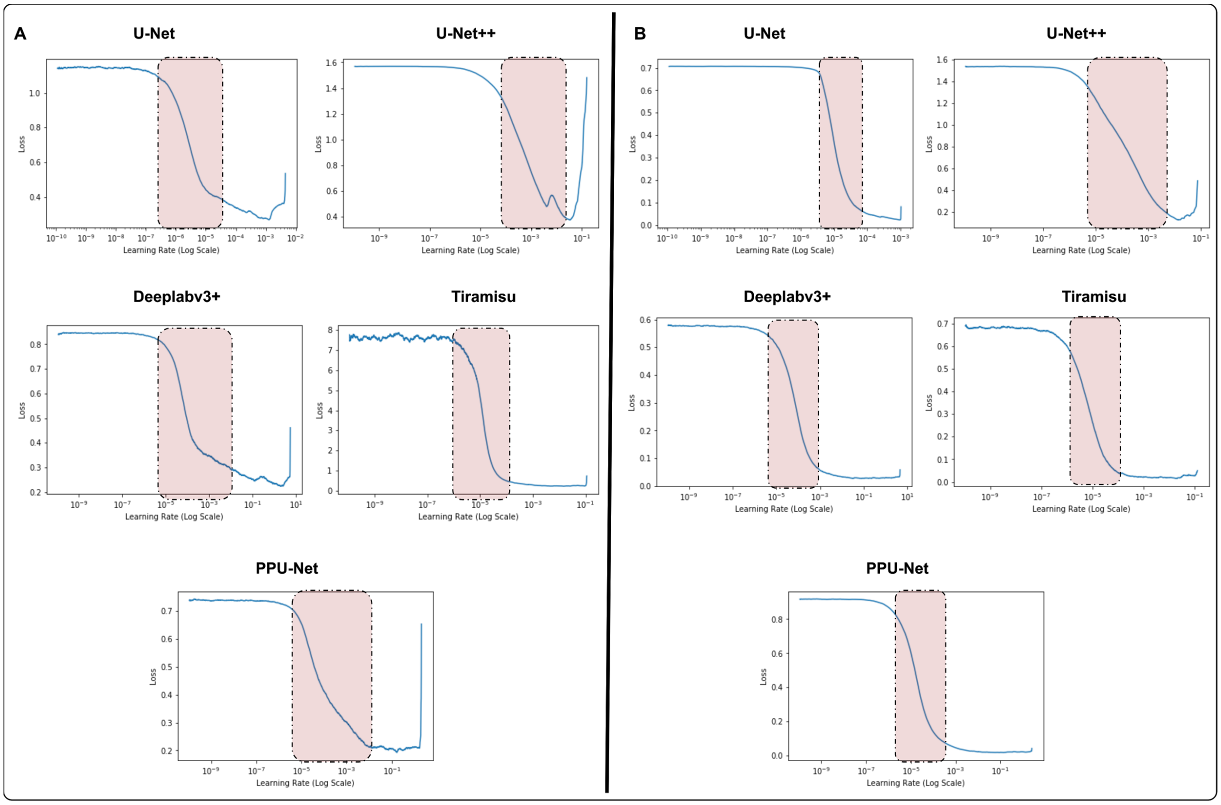

Figure S2: Picking a learning rate. (A) Model loss (y-axis) at different learning rate values (x-axis) for different models (panels) on brightfield data. The best learning rate is selected from the steep area in

the curve (manually selected shaded box; Methods). (B) As (A), but for fluorescence data.

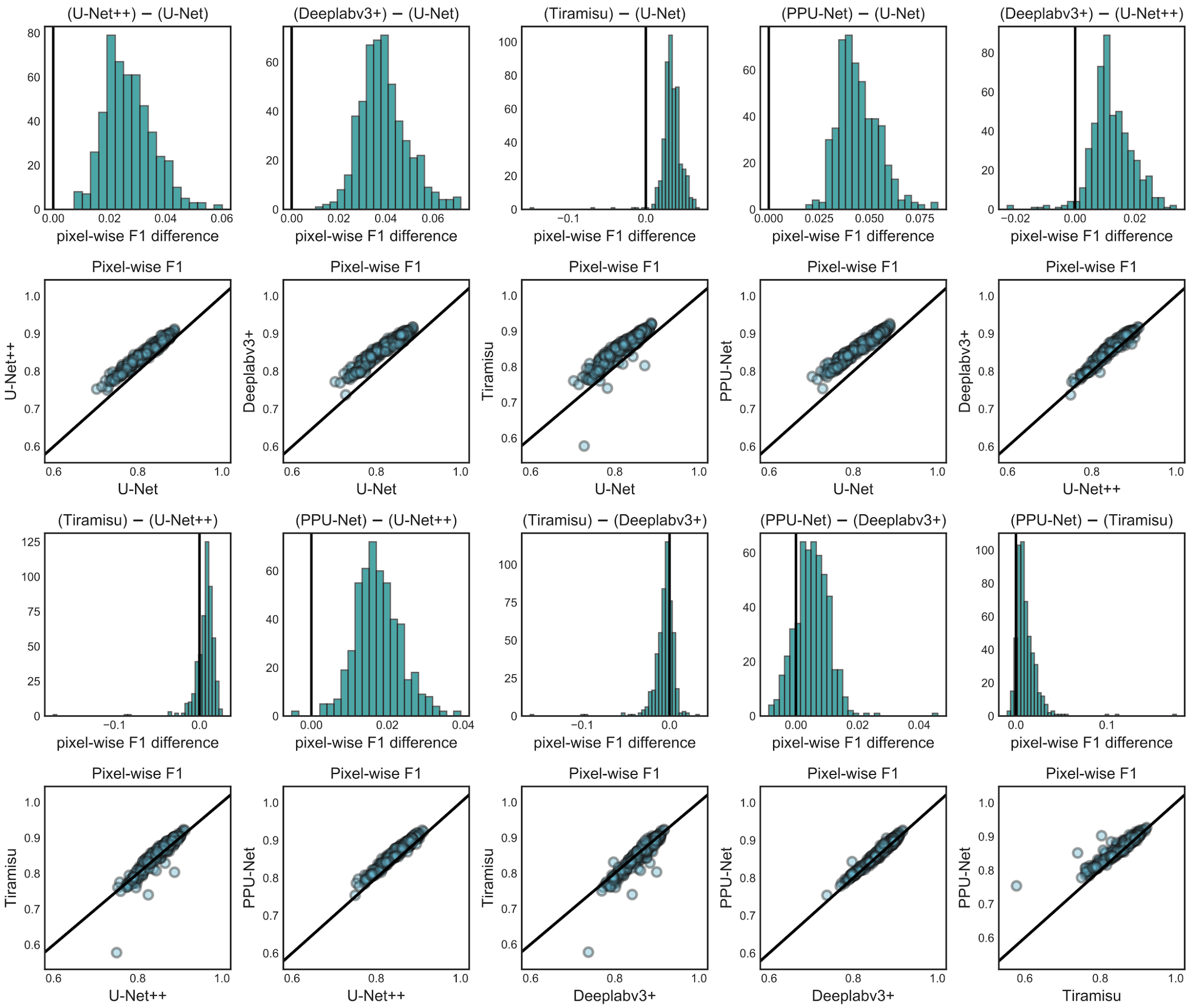

Figure S3: Pairwise comparison of pixel-wise model performance using the seven cell lines data. Scatter plots: Per-image average pixel-wise F1 score (markers) for two models (x- and y-axis) for pairs of models (panels). Solid black line: y = x (equal performance). Histograms: Frequency (y-axis) of per-image performance difference (x-axis) for the same model pairs (panels). Solid black line: x = 0 (equal performance).

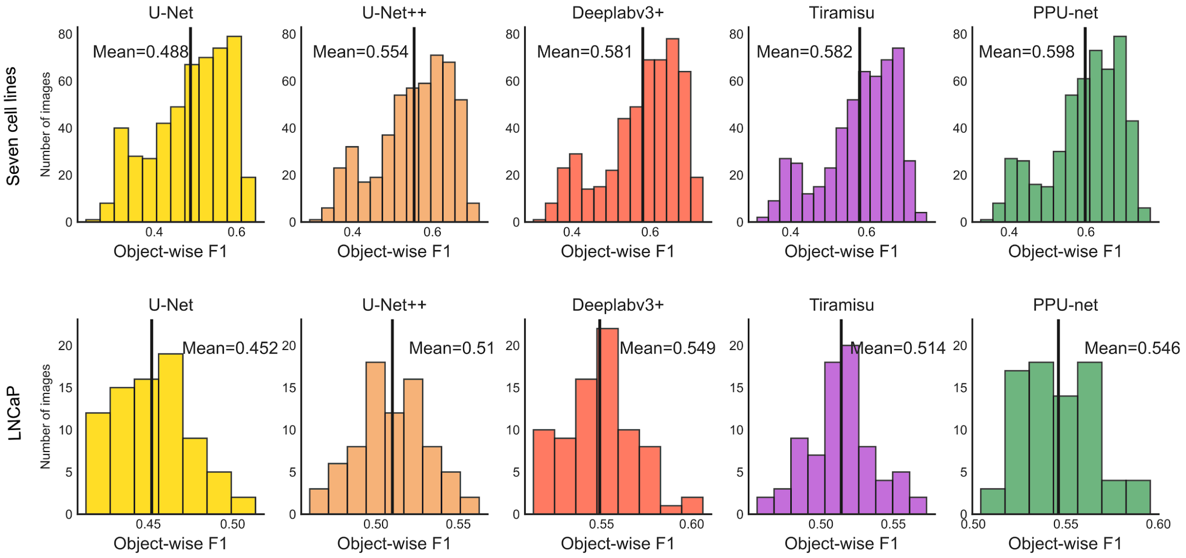

Figure S4: Frequency (y-axis) of IoU-average (across 0.5 to 0.95 cutoffs as in Caicedo *et al.*) per-image object-wise F1 scores (x-axis) for the considered models (colors, panels) in seven cell lines (top) and LNCaP datasets(bottom). Black line and label: average value.

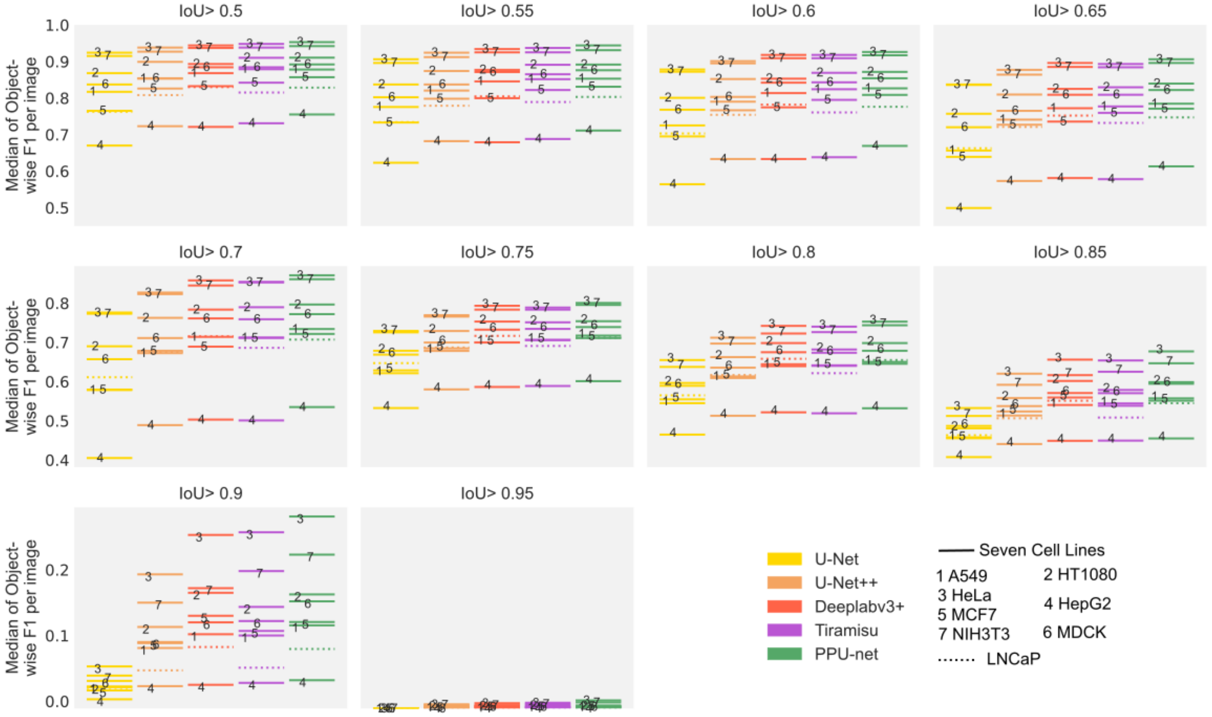

Figure S5: Per-image median object-wise F1 scores (y-axis) across seven cell lines (solid lines), and LNCaP (dotted lines) for all models (colors) and a range of IoU thresholds (panels; Methods).

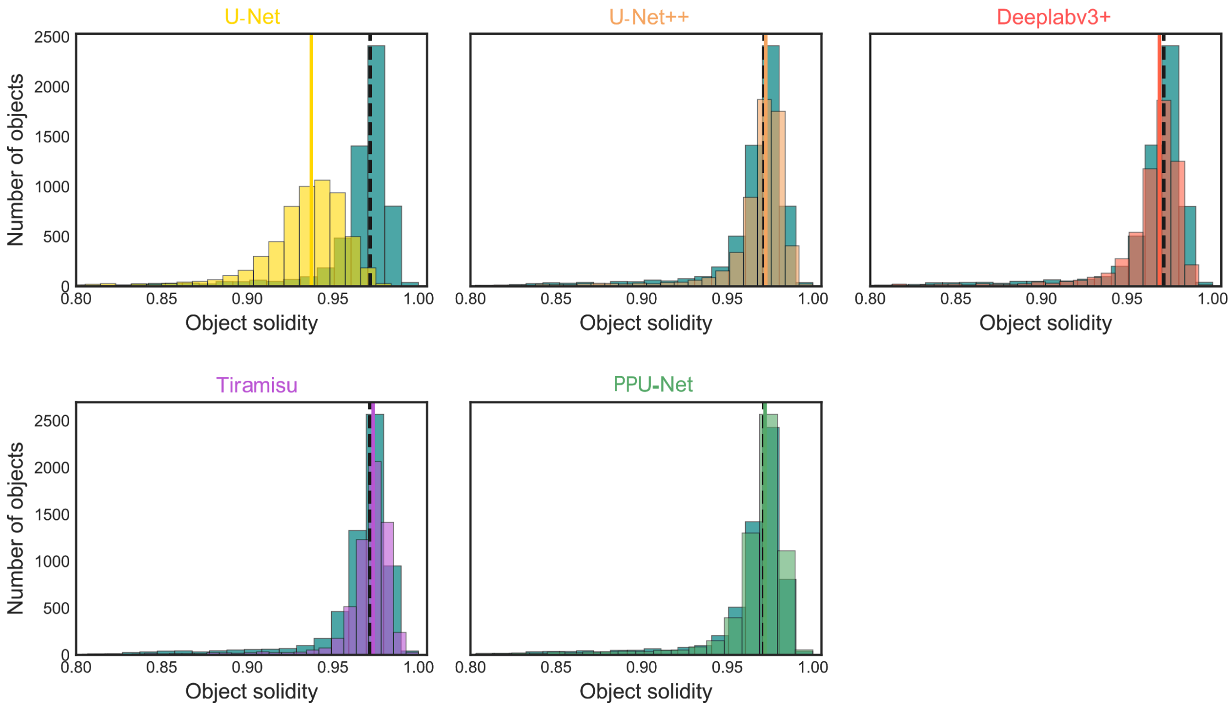

Figure S6: Frequency (y-axis) object solidity (ratio between the number of pixels in an object to the number of pixels in the convex hull of the object; x-axis) for each model (colors; panels), and the ground truth (teal) for 20 images randomly sampled from the test set. A) U-Net, B) U-Net++, C) Deeplabv3+, D) Tiramisu, E) PPU-Net.

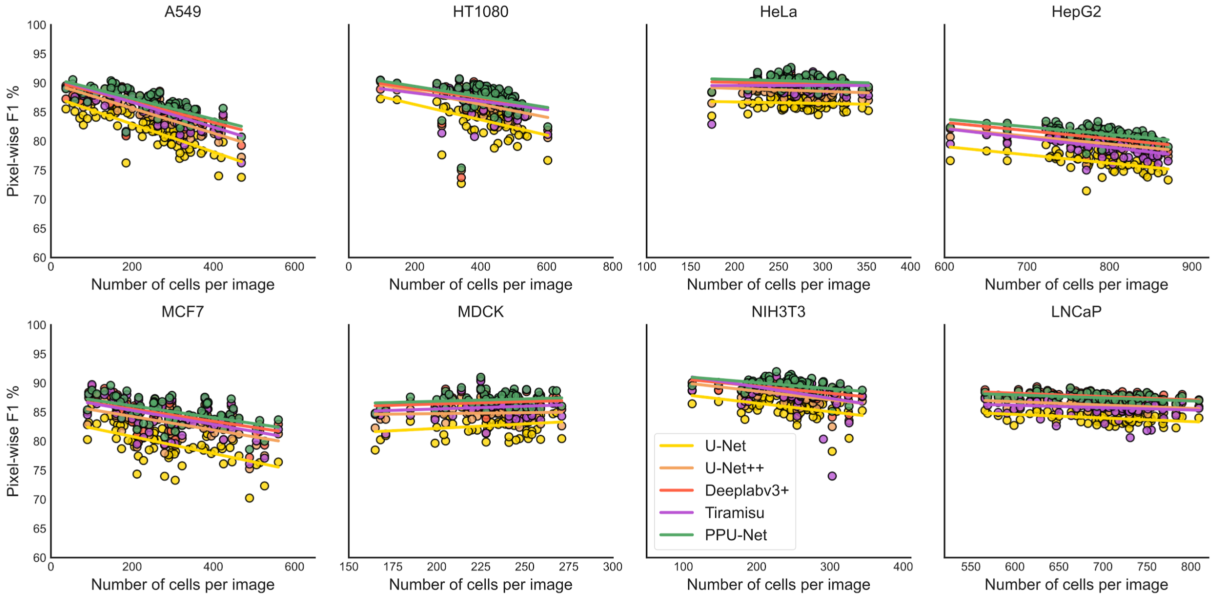

Figure S7: Model performance in images with different nuclei densities. Pixel-wise F1 score (y-axis) at different numbers of cells per image (x-axis) for individual images (markers) using different models (colors) on the various cell lines (panels). Solid lines: regression fit.

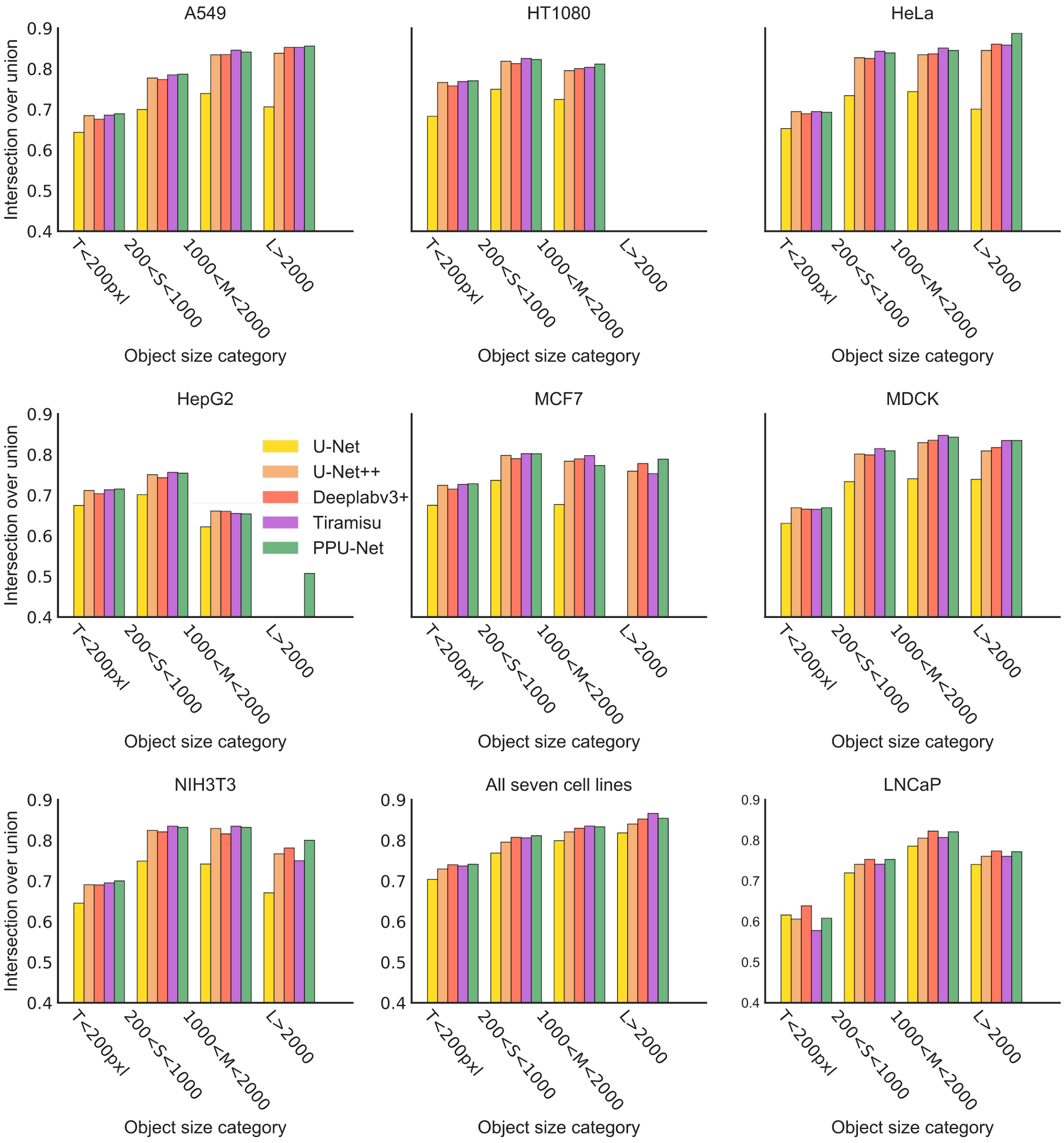

Figure S8: Impact of nuclear size on model performance. Intersection over union metric ("IoU", y-axis) for four size categories of nuclei (x-axis) for different models (colors) and cell lines (panels). Labels: Tiny (T), Small (S), Medium (M), and Large (L).

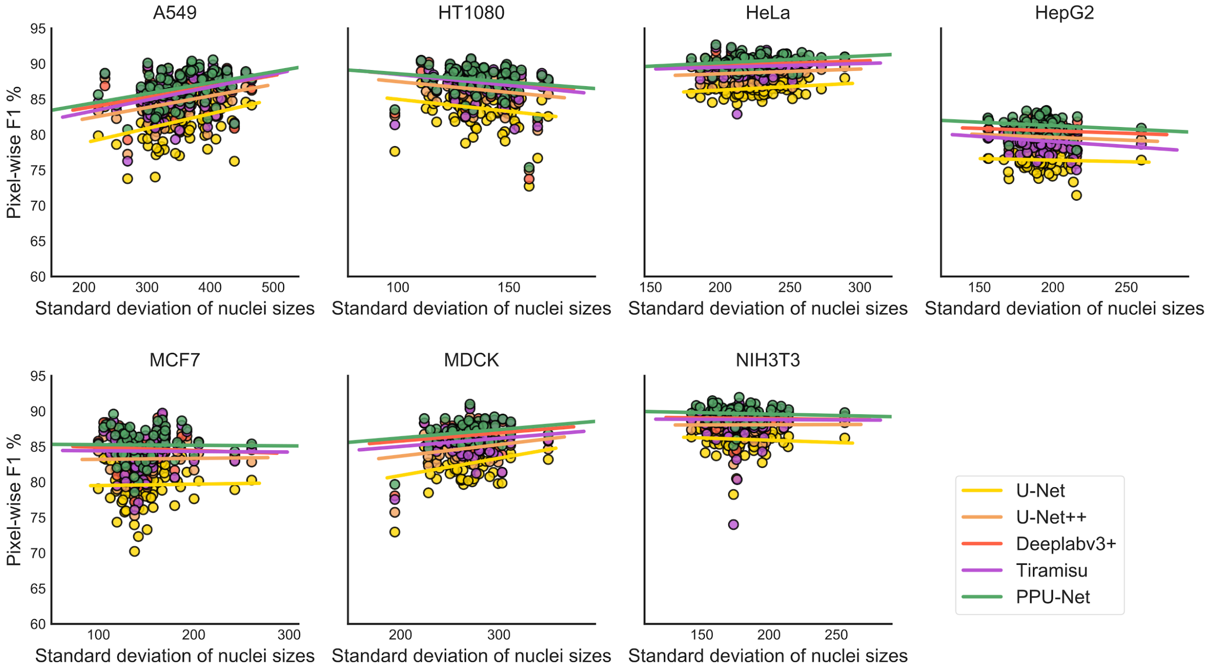

Figure S9: Impact of nucleus size variation on model performance. Pixel-wise F1 score (y-axis) for individual images (markers) contrasted against the standard deviation of nucleus sizes in the image (x-axis) for different models (colors) and cell lines (panels). Solid lines: regression fit.

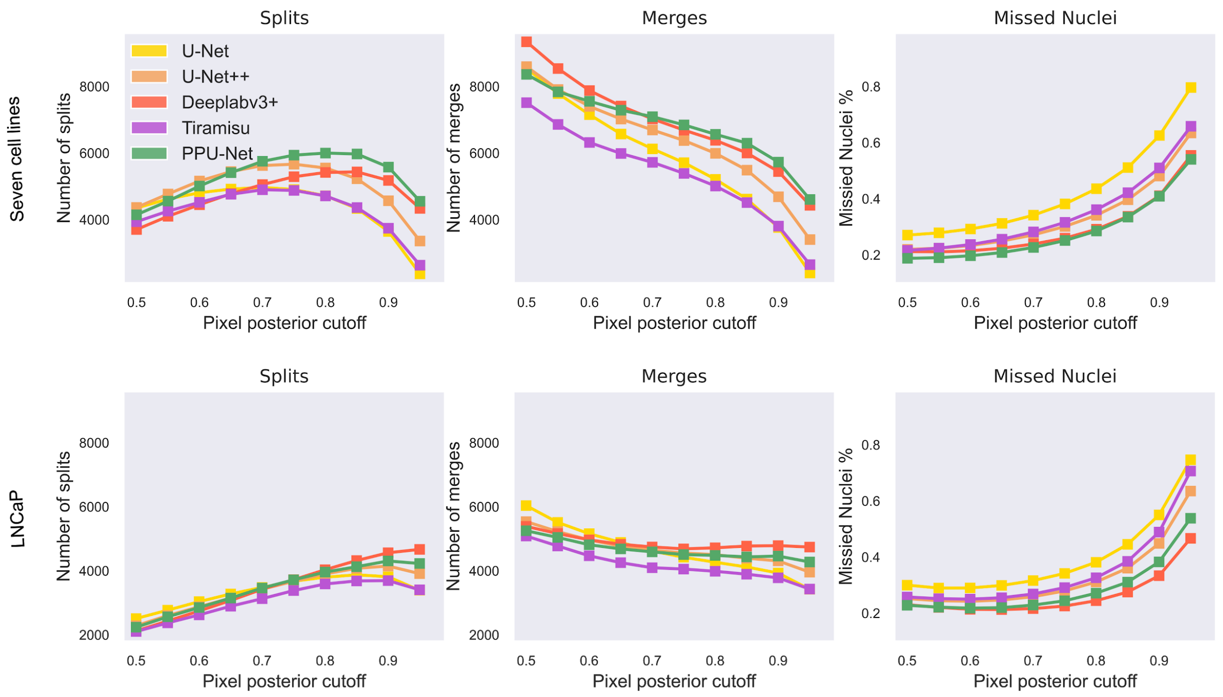

Figure S10: Object-level errors at different prediction probability thresholds. Number of errors (y-axis) at a range of pixel posterior cutoffs (x-axis) with different models (colors) and different datasets (seven cell lines top and LNCaP bottom). (A) Splits (B) Merges (C) Missed nuclei.

Table S1: Average number of cells in a field of view (FOV) in different cell lines (rows) and different data splits (columns)

Cell Line	Dataset	Average nuber of cells per FOV in train split	Average nuber of cells per FOV in validation split	Average nuber of cells per FOV in test split
A549	Seven cell lines	233	327	245
HT1080	Seven cell lines	389	338	415
HeLa	Seven cell lines	227	348	273
HepG2	Seven cell lines	778	378	778
MCF7	Seven cell lines	306	317	290
MDCK	Seven cell lines	237	389	230
NIH3T3	Seven cell lines	244	484	244
LNCaP	LNCaP	677	690	701

Table S2: Gain in percent in F1-score on held-out data of using label smoothing for different models and different smoothing factors.

Model	Smoothing Factor	Training Images Per Cell Line						
		1	2	4	8	16	32	64
U-Net	0.05	1.610	3.852	6.713	-0.277	-1.51	-1.131	-1.017
U-Net	0.10	-0.798	3.320	5.486	-1.023	-2.871	-2.092	0.573
U-Net	0.15	0.933	2.241	5.779	-0.808	-1.284	-6.725	-1.012
U-Net	0.20	1.196	2.658	4.988	1.376	-0.787	-3.477	0.428
U-Net++	0.05	1.911	0.809	0.566	-0.190	0.205	-0.0180	0.130
U-Net++	0.10	2.047	0.576	0.037	-0.279	0.430	-0.186	0.099
U-Net++	0.15	2.43	0.347	0.701	0.290	0.290	-0.107	-0.111
U-Net++	0.20	2.212	0.460	0.612	-0.251	0.283	-0.095	-0.208
Deeplabv3+	0.05	-1.050	2.360	-1.554	-9.431	-0.011	0.576	1.378
Deeplabv3+	0.10	-0.693	2.251	-0.358	-6.075	-5.045	-1.399	-2.792
Deeplabv3+	0.15	0.344	2.865	-1.717	-9.14	-8.703	-16.895	-6.437
Deeplabv3+	0.20	-0.569	2.017	-1.623	-3.832	-1.395	1.729	-9.737
Tiramisu	0.05	16.133	-0.054	3.313	1.098	3.526	1.008	-0.337
Tiramisu	0.10	17.061	3.663	2.824	0.52	3.096	1.965	0.012
Tiramisu	0.15	17.762	3.807	3.772	1.670	3.501	2.224	0.440
Tiramisu	0.20	19.912	3.914	2.733	1.852	3.275	0.927	-0.283
PPU-Net	0.05	0.158	0.283	0.043	0.407	0.481	0.26	-0.476
PPU-Net	0.10	2.986	0.771	0.123	-0.227	0.570	-0.006	0.512
PPU-Net	0.15	3.957	0.103	0.437	0.143	0.080	0.187	0.141
PPU-Net	0.20	2.459	0.419	0.373	-0.161	0.644	0.180	-0.018

Table S3: Joint models segmentation pixel- and object-wise F1-score for different cell lines of fluorescent modality.

cell_line	U-Net		U-Net++		Deeplabv3+		Tiramisu		PPU-Net	
	PW	OW	PW	OW	PW	OW	PW	OW	PW	OW
A549	0.987	0.967	0.989	0.971	0.984	0.961	0.987	0.97	0.989	0.972
HT1080	0.988	0.97	0.99	0.975	0.984	0.964	0.987	0.973	0.99	0.976
HeLa	0.988	0.973	0.99	0.975	0.985	0.968	0.988	0.975	0.99	0.975
HepG2	0.982	0.938	0.985	0.951	0.978	0.91	0.982	0.945	0.986	0.956
MCF7	0.983	0.951	0.986	0.96	0.98	0.936	0.981	0.955	0.987	0.964
MDCK	0.987	0.965	0.99	0.969	0.985	0.964	0.987	0.968	0.989	0.97
NIH3T3	0.991	0.978	0.992	0.98	0.988	0.976	0.991	0.979	0.992	0.98

Table S4: Models segmentation pixel-wise (PW) and object-wise (OW) F1-score for different cell lines of brightfield modality in both the seven cell lines and the LNCaP datasets.

cell_line	U-Net		U-Net++		Deeplabv3+		Tiramisu		PPU-Net	
	PW	OW	PW	OW	PW	OW	PW	OW	PW	OW
A549	0.803	0.441	0.84	0.53	0.854	0.561	0.85	0.562	0.859	0.576
HT1080	0.827	0.501	0.861	0.588	0.873	0.616	0.867	0.608	0.873	0.617
HeLa	0.86	0.571	0.889	0.657	0.901	0.691	0.896	0.685	0.903	0.699
HepG2	0.758	0.318	0.796	0.388	0.805	0.396	0.794	0.399	0.812	0.414
MCF7	0.781	0.43	0.83	0.522	0.843	0.551	0.84	0.548	0.847	0.559
MDCK	0.818	0.478	0.851	0.554	0.868	0.596	0.857	0.588	0.869	0.602
NIH3T3	0.851	0.56	0.88	0.639	0.89	0.662	0.884	0.66	0.894	0.677
LNCaP	0.840	0.452	0.862	0.5109	0.877	0.549	0.858	0.514	0.874	0.546
